# Relationship of National Institutes of Health Stroke Scale to 30-Day Mortality in Medicare Beneficiaries With Acute Ischemic Stroke

**DOI:** 10.1161/JAHA.111.000034

**Published:** 2012-02-20

**Authors:** Gregg C. Fonarow, Jeffrey L. Saver, Eric E. Smith, Joseph P. Broderick, Dawn O. Kleindorfer, Ralph L. Sacco, Wenqin Pan, DaiWai M. Olson, Adrian F. Hernandez, Eric D. Peterson, Lee H. Schwamm

**Affiliations:** Division of Cardiology, University of California, Los Angeles, CA (G.C.F.); Department of Neurology, University of California, Los Angeles (J.L.S.); Department of Clinical Neurosciences, University of Calgary, Alberta, Canada (E.E.S.); Department of Neurology, University of Cincinnati Academic Health Center, OH (J.P.B., D.O.K.); Miller School of Medicine, University of Miami, FL (R.L.S.); Duke Clinical Research Center, Durham, NC (W.P., D.M.O., A.F.H., E.D.P.); Division of Neurology, Massachusetts General Hospital, Boston (L.H.S.)

**Keywords:** ischemic stroke, National Institutes of Health Stroke Scale, mortality, registries

## Abstract

**Background:**

The National Institutes of Health Stroke Scale (NIHSS), a well-validated tool for assessing initial stroke severity, has previously been shown to be associated with mortality in acute ischemic stroke. However, the relationship, optimal categorization, and risk discrimination with the NIHSS for predicting 30-day mortality among Medicare beneficiaries with acute ischemic stroke has not been well studied.

**Methods and Results:**

We analyzed data from 33102 fee-for-service Medicare beneficiaries treated at 404 Get With The Guidelines-Stroke hospitals between April 2003 and December 2006 with NIHSS documented. The 30-day mortality rate by NIHSS as a continuous variable and by risk-tree determined or prespecified categories were analyzed, with discrimination of risk quantified by the *c*-statistic. In this cohort, mean age was 79.0 years and 58% were female. The median NIHSS score was 5 (25th to 75th percentile 2 to 12). There were 4496 deaths in the first 30 days (13.6%). There was a strong graded relation between increasing NIHSS score and higher 30-day mortality. The 30-day mortality rates for acute ischemic stroke by NIHSS categories were as follows: 0 to 7, 4.2%; 8 to 13, 13.9%; 14 to 21, 31.6%; 22 to 42, 53.5%. A model with NIHSS alone provided excellent discrimination whether included as a continuous variable (*c*-statistic 0.82 [0.81 to 0.83]), 4 categories (*c*-statistic 0.80 [0.79 to 0.80]), or 3 categories (*c*-statistic 0.79 [0.78 to 0.79]).

**Conclusions:**

The NIHSS provides substantial prognostic information regarding 30-day mortality risk in Medicare beneficiaries with acute ischemic stroke. This index of stroke severity is a very strong discriminator of mortality risk, even in the absence of other clinical information, whether used as a continuous or categorical risk determinant. **(*J Am Heart Assoc*. 2012;1:42-50.)**

Stroke is among the leading causes of death, disability, hospitalizations, and healthcare expenditures in the United States.^1^ National quality profiling efforts have recently begun to report hospital-level performance for Medicare beneficiaries, including 30-day mortality rates, for common medical conditions including heart failure, acute myocardial infarction, and community-acquired pneumonia.^2–5^ There is growing interest in also reporting outcomes for Medicare beneficiaries hospitalized with acute ischemic stroke.^6^ Risk adjustment is critical for accurately assessing and reporting clinical outcomes.^2–4^ Stroke severity on admission is a powerful determinate of functional outcomes in acute ischemic stroke.^7–9^ The National Institutes of Health Stroke Scale (NIHSS), which is a validated tool for assessing the initial stroke severity, has been shown to predict mortality in acute ischemic stroke in several prior studies.^9–14^ However, these studies have generally been confined to small numbers of patients from single centers, select patients enrolled in randomized clinical trials, studies outside the United States, or limited to evaluating in-hospital mortality. The relation of admission NIHSS to 30-day mortality and its capability to discriminate risk in Medicare beneficiaries with acute ischemic stroke have not be well studied. Using data from ischemic stroke admissions in the Get With The Guidelines-Stroke (GWTG-Stroke) Program linked to Medicare data, the objectives of this study were to (1) quantify the relation and risk discrimination of admission NIHSS to 30-day mortality rates among Medicare beneficiaries hospitalized with acute ischemic stroke and (2) identify categories of NIHSS that provide optimal discrimination of 30-day mortality risk.

## Methods

### Data Sources

Data from the GWTG-Stroke registry was linked with enrollment files and inpatient claims from the Centers for Medicare & Medicaid Services (CMS) for the period April 1, 2003, through December 31, 2006. Follow-up continued through 2007. The design, inclusion criteria, and data collection methods for GWTG-Stroke have been described previously.^15,16^ Patients were eligible for inclusion in the GWTG-Stroke registry if they were admitted for acute stroke. Trained hospital personnel ascertained acute ischemic stroke admissions by either prospective clinical identification, retrospective identification using *International Classification of Diseases (ICD)*-9 discharge codes, or a combination. Patient data abstracted by trained hospital personnel included demographics, medical history, brain imaging, in-hospital treatment and events, discharge treatment and counseling, mortality, and discharge destination. All patient data were de-identified before submission. All states and regions of the United States were represented and a variety of centers participated, from community hospitals to large tertiary centers. Data on hospital-level characteristics (ie, bed size, academic or nonacademic status, and geographic region) were obtained from the American Hospital Association.^17^ All participating institutions were required to comply with local regulatory and privacy guidelines and, if applicable, to obtain institutional review board approval. Outcome Sciences, Inc (Cambridge, MA) served as the registry-coordinating center. The Duke Clinical Research Institute (Durham, NC) served as the data analysis center.

The CMS files (100% Medicare research identifiable files) included data for all fee-for-service Medicare beneficiaries aged ≥65 years who were hospitalized with a diagnosis of acute stroke (*ICD-9-* 430.x, 431.x, 433.x, 434.x, and 436.x). We merged patient data in the GWTG registry with Medicare Part A inpatient claims, matching by admission and discharge dates, hospital, date of birth, and sex using methods previously described.^16,18^ From 222278 hospitalizations of patients aged 65 years or older in GWTG-Stroke, we matched 157039 patients (69%) to fee-for-service Medicare claims from 850 hospitals. Patients in Medicare–managed care plans (15% to 25% of the population depending on the region of the country) or other types of insurance are not included in fee-for-service Medicare claims files and therefore cannot be matched.^16,18^ The study population was further confined to patients with acute ischemic stroke (N=101801), first index stroke admission April 2003 or later, and the first index admission (N=94421). Hospitals and patients from centers with <25 ischemic stroke patients entered during the study period were excluded to minimize the likelihood of sampling error. This resulted in a study population of 91134 acute ischemic stroke patients from 625 GWTG-Stroke hospitals.

The NIHSS score was documented in 33770 of these patients (37.1%) from 560 hospitals. For additional stability at the hospital level, we further confined the analysis to hospitals with at least 10 patients with NIHSS documented during the study period. This resulted in a final study population of 33102 patients from 404 participating hospitals. The demographics, clinical characteristics, and geographic distribution of matched and unmatched acute ischemic stroke cases were similar, except fewer patients were enrolled in GWTG-Stroke from the Midwest compared with unmatched ischemic stroke cases. The demographics, clinical characteristics, and geographic distribution of patients with and without NIHSS recorded were similar. The 30-day mortality was modestly lower in the 33102 patients with NIHSS recorded (13.6%) compared with the 57364 patients without NIHSS recorded (14.6%), *P*<0.0001.

### National Institutes of Health Stroke Scale

The NIHSS is a 15-item neurologic examination stroke scale used to evaluate the effect of acute ischemic stroke on the levels of consciousness, language, neglect, visual-field loss, extraocular movement, motor strength, ataxia, dysarthria, and sensory loss that provides a quantitative measure of stroke-related neurologic deficit.^7^ The scale is designed to be a simple, valid, and reliable tool that can be administered at the bedside consistently by physicians, nurses, or therapists. A trained observer rates the patient's ability to answer questions and perform activities. Ratings for each item are scored with 3 to 5 grades with 0 as normal. The NIHSS can be viewed online at http://www.ninds.nih.gov/doctors/NIH_Stroke_Scale.pdf.

The first recorded NIHSS score, as close to admission as possible, was collected when available. Stroke team members were highly encouraged, but not required, to complete training and certification in the NIHSS. There were 2 patients (0.006%) with NIHSS score recorded with a value >42 who were not included in the analyses. There were also a few patients with improbable NIHSS scores of 41 (N=14) (0.04%) and 42 (N=16) (0.05%). Although NIHSS scores of 41 and 42 should be unobtainable among patients who are alive and most likely reflect slight scale errors in patients with severe stroke severity, these NIHSS scores were analyzed as recorded.

### Outcome

The outcome of interest was all-cause mortality within 30 days from time of admission. As with other studies of Medicare beneficiaries, we obtained, with complete ascertainment, deaths and dates of death from the CMS vital status files.^16,18^

### Statistical Analysis

We summarized baseline characteristics by whether the patient was alive or dead at 30 days using percentages for categorical variables and means and SDs for continuous variables. For comparisons by group, we used chi-square tests for categorical variables and Wilcoxon tests for continuous variables. Frequency histograms were used to describe the distribution of NIHSS scores. NIHSS was analyzed as a continuous variable as well as in categorical groups. We used classification and regression tree methodology^19^ to determine the optimal cut points for 30-day mortality by categorical grouping of NIHSS ranging from 2 to 6 groups. We also analyzed a 3-category grouping of the NIHSS found in a prior study to discriminate 6-month dependency outcomes.^8^ Discrimination by NIHSS as a continuous variable and by categorical groups was assessed by determining Wald chi square and the *c* statistic. A *c* statistic of 1.0 indicates perfect prediction whereas a *c* statistic of 0.50 indicates prediction no better than chance alone. The observed versus predicted mortality by NIHSS as a continuous variable for 10 deciles of expected risk was plotted. Discrimination by NIHSS as a continuous variable was also examined among several clinically relevant subgroups. A previously derived and validated GWTG-Stroke clinical risk prediction model for acute ischemic stroke was used to evaluate the discrimination of a clinical model without and with NIHSS added.^16^ This model included patient characteristics (age, sex, race/ethnicity, medical history of previous stroke or transient ischemic attack, coronary heart disease [CAD], myocardial infarction, carotid stenosis, chronic obstructive pulmonary disease, dementia, diabetes, peripheral vascular disease, pneumonia, renal dysfunction, and hypertension), on-hour arrival time to hospital (Monday through Friday 7 am to 5 pm, yes/no), and Emergency Medical Service transport. SAS software version 9.2 (SAS Institute Inc, Cary, NC) was used for all analyses.

## Results

**Figure 1.**
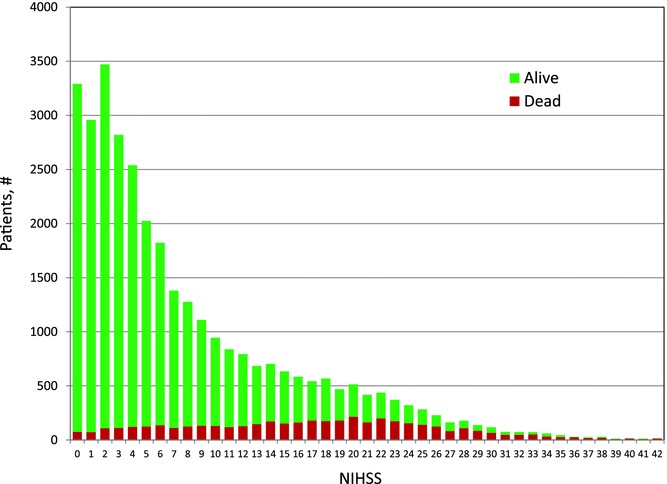
Distribution of NIHSS scores among Medicare beneficiaries in GTWG-Stroke hospitals with acute ischemic stroke patients alive at 30 days are shown in green and patients dead by 30 days shown in red. NIHSS indicates National Institutes of Health Stroke Scale; GWTG, Get With The Guidelines.

There were 33102 acute ischemic stroke patients with NIHSS documented in the study cohort. The patient characteristics are shown in Table 1. The median age was 79 years, 56.5% were women, and 83.3% were white. Prior stroke or transient ischemic attack was present in 30.0% of patients. Comorbidities were common with hypertension in 77.5%, diabetes in 26.7%, coronary heart disease in 32.4%, and history of atrial fibrillation or flutter in 24.5%. The NIHSS median score in this population was 5 (25th to 75th percentile 2 to 12). The distribution of NIHSS scores in this patient population are shown in Figure 1. Close to two thirds of patients were reported to have an admission NIHSS score of ≤8. Of the 404 GWTG-Stroke hospitals included in this study, median bed size was 409, all regions of the United States were represented, and nonacademic hospitals accounted for 36.8% of admissions (Table 1).

**Table 1.** Patient and Hospital Characteristics of Medicare Beneficiaries in GTWG-Stroke Hospitals With Acute Ischemic Stroke Overall and by Alive or Dead at 30 Days

Variable	Overall Cohort (N=33102)	No Death Within 30 Days (N=28606)	Death Within 30 Days (N=4496)	*P* Value*
Age, y, median	79	79	83	<0.0001

25th–75th	73–85	72–84	78–88	

Sex, male, %	43.6	44.3	38.9	<0.0001

Race/ethnicity, %				<0.0001

Hispanic	2.5	2.6	1.9	

Black	8.7	9.1	5.8	

White	83.3	82.7	87.1	

Arrival mode, %				<0.0001

Emergency medical services	67.6	63.9	91.4	

Private transport	32.4	36.1	8.6	

Arrival time, off hours, %	53.4	53.3	54.3	0.1930

Atrial fibrillation/flutter, %	24.5	22.0	40.9	<0.0001

Previous stroke/TIA, %	30.0	29.8	31.6	0.0156

CAD/prior MI, %	32.4	31.6	37.8	<0.0001

Diabetes mellitus, %	26.7	26.8	25.7	0.1267

Peripheral vascular disease, %	5.5	5.3	6.7	0.0003

Hypertension, %	77.5	77.7	76.3	0.0293

Smoker, %	11.3	11.8	8.4	<0.0001

Dyslipidemia, %	36.4	37.9	26.8	<0.0001

Body mass index, kg/m^2^	25.8	26.0	24.4	<0.0001

25th–75th	22.8–30.0	23.0–29.8	21.5–28.0	

NIHSS total score mean, (SD)	8.01 (7.88)	6.62 (6.67)	16.85 (9.18)	<0.0001

Median	5	4	17	

25th–75th	2–12	2–9	10–23	

Discharge status, %				

Home	36.6	41.9	2.9	<0.0001

Skilled nursing facility	24.0	24.5	20.4	

Rehabilitation, %	26.7	30.2	4.7	

Transfer to acute care facility	2.3	2.5	1.4	

Left against medical advice	0.2	0.2	0.2	

Hospice	3.8	0.6	24.7	

Died inhospital	6.4	-	45.8	

Ambulatory status†				<0.0001

Ambulate independently	39.1	44.4	3.7	

With assistance	34.3	37.8	10.1	

Unable to ambulate	24.2	15.9	79.4	

Hospital characteristic				

No. of stroke discharges, %				0.8495

0–100	9.9	9.9	9.7	

101–300	57.4	57.4	57.3	

>300	32.7	32.7	33.0	

No. of beds, median	409	409	414	0.6929

25th–75th	279–580	279–580	279–580	

Geographic region, %				0.9551

Northeast	25.8	25.7	26.0	

Midwest	22.4	22.4	22.3	

South	36.4	36.4	36.1	

West	15.5	15.5	15.6	

Hospital type, %				0.7908

Academic	63.2	63.2	63.4	

Nonacademic	36.8	36.9	36.6	

Missing	0.04	0.04	0.02	

TJC primary stroke center, %	74.7	74.7	74.8	0.8329

GWTG indicates Get With The Guidelines; TIA, transient ischemic attack; CAD/prior MI, coronary artery disease or myocardial infarction; NIHSS, National Institutes of Health Stroke Scale; SD, standard deviation; TJC, The Joint Commission.

* Wilcoxon 2-sample test for continuous variables and chi-square test for categorical variables.

† Excludes patients who transferred out.

There were 4496 deaths within the first 30 days (13.6%). There were 2037 deaths that occurred during the index hospitalization (inhospital mortality 6.4%). The patient and hospital characteristics of those patients who died in the first 30 days compared with those who were alive are shown in Table 1. Patients who died within 30 days from admission were older, female, more likely to be white, had a higher frequency of atrial fibrillation, and prior history of coronary artery disease (CAD). Patients who died were more likely to have arrived by emergency medical services (EMS). The median NIHSS was substantially higher among patients who died compared with those who were alive at 30 days (17 versus 4, *P*<0.0001). There were no significant differences in hospital characteristics between patients who were alive or dead in the first 30 days.

**Figure 2.**
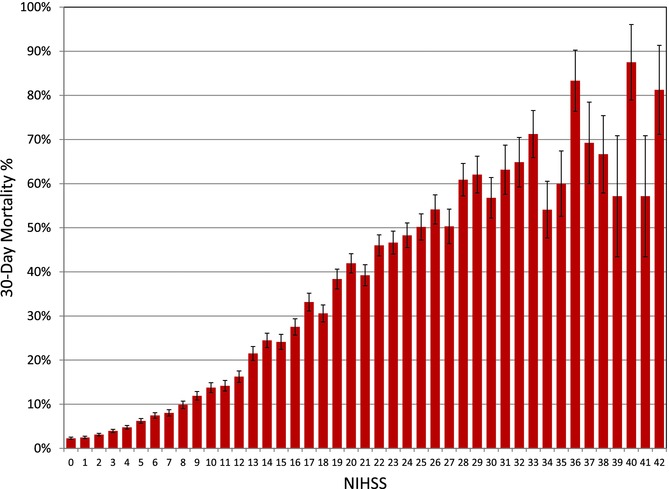
Acute ischemic stroke 30-day mortality rates by NIHSS. Standard error bars are displayed. NIHSS indicates National Institutes of Health Stroke Scale.

**Figure 3.**
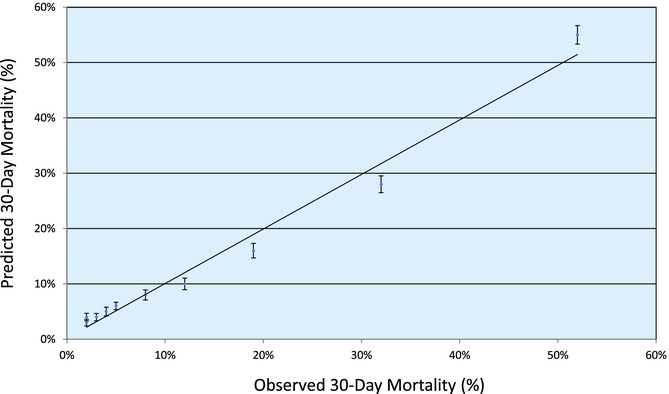
Calibration plot of observed and predicted 30-day mortality by NIHSS as a continuous variable.

The relation between each score on the NIHSS and 30-day mortality is shown in Figure 2. There was a graded near-linear relationship between increases in the NIHSS and 30-day mortality. The 30-day mortality rate was 2.3% for patients with a score of 0 and was >75% for patients with a score of ≥40. As a continuous variable, NIHSS provided excellent discrimination of 30-day mortality with a *c*-statistic 0.82, (95% confidence interval [CI], 0.81–0.83). A calibration plot of observed and expected 30-day mortality with NIHSS as a continuous variable is shown in Figure 3. Categorizations of NIHSS by risk-tree methodology with optimal cut points for 30-day mortality for different group sizes are shown in Table 2. There was slightly less discrimination with these categorical grouping with 4 or more groups compared with NIHSS as a continuous variable, with the risk-tree methodology selecting a categorization with 4 groups and cut points of 0 to 7, 8 to 13, 14 to 21, and 22 to 42. This allows identification on admission of acute ischemic stroke patients at low (<10%), medium (10% to 20%), high (20% to 40%), and very high (>40%) 30-day mortality risk as shown in Figure 2 and Table 3. The *c*-statistic with this 4-group categorical approach to NIHSS was 0.80 (95% CI, 0.79–0.80). The 30-day mortality rates for these 4 NIHSS categories 0 to 7, 8 to 13, 14 to 21, and 22 to 42 were 4.2%, 13.9%, 31.6%, and 53.5%, respectively (Table 3). Although patients with an NIHSS score of 22 to 42 constitute only 8.2% of the cohort with acute ischemic stroke, these patients account for close to one third of all deaths. Alternative 3 group categories for NIHSS are shown in Table 3. A categorization of patients with mild 0 to 5, moderate 6 to 13, and severe 14 to 42 stroke severity by NIHSS produced groups with 30-day mortality rates of 3.6%, 11.6%, and 39.9% and a *c*-statistic of 0.79 (95% CI, 0.78–0.80).

**Table 2.** Categorical Groupings of NIHSS to Best Discriminate 30-Day Mortality Risk

No. of Groupings	Risk-Tree Cut Points for the Groupings	Chi-Square for 30-Day Mortality	*c*-Statistic (95% Confidence Intervals)
2	0–13, 14–42	5378	0.74 (0.74–0.75)

3	0–13, 14–21, 22–42	6069	0.75 (0.75–0.76)

4	0–7, 8–13, 14–21, 22–42	6418	0.80 (0.79–0.80)

5	0–7, 8–13, 14–18, 19–21, 22–42	6541	0.80 (0.79–0.81)

6	0–7, 8–13, 14–18, 19–21, 22–27, 28–42	6651	0.80 (0.79–0.81)

Original (NIHSS as continuous variable)	0, 1, 2, 3, 4, …., 42	6856	0.82 (0.81–0.83)

NIHSS indicates National Institutes of Health Stroke Scale.

Classification and regression tree methodology (in R) with predetermined No. of nodes were applied to identify the categories of NIHSS. Larger chi-square statistic and *c*-statistic closer to 1.0 indicates more optimal discrimination of risk.

**Table 3.** Relation of NIHSS Categories to 30-Day Mortality in Acute Ischemic Stroke

NIHSS Categories	30-Day Mortality	Portion of Patients	Portion of Deaths
0–7	4.2% (4.0%–4.5%)	61.3%	19.2%

8–13	13.9% (13.0%–14.8%)	17.1%	17.4%

14–21	31.6% (30.2%–32.9%)	13.4%	31.1%

22–42	53.5% (51.7%–55.4%)	8.2%	32.3%

*c*-statistic 0.80 (0.79–0.80)			

0–7	4.2% (4.0%–4.5%)	61.3%	19.2%

8–13	13.9% (13.0%–14.8%)	17.1%	17.4%

14–42	39.9% (38.8%–41.0%)	21.6%	63.4%

*c*-statistic 0.79 (0.78–0.79)			

0–5*	3.6% (3.3%–3.9%)	51.7%	13.7%

6–13*	11.6% (11.0%–12.3%)	26.7%	22.9%

14–42*	39.9% (38.8%–41.0%)	21.6%	63.4%

*c*-statistic 0.79 (0.78–0.80)			

NIHSS indicates National Institutes of Health Stroke Scale.

* This categorization derived from reference 8.

The 95% confidence intervals are shown in parentheses.

NIHSS provided excellent discrimination of 30-day mortality for patients 65 to 79 years of age and those 80 or older, men and women, and patients with and without prior stroke or transient ischemic attack (Table 4). The clinical risk model for Medicare beneficiaries based on demographics and clinical variables without NIHSS had more modest discrimination (*c*-statistic 0.71 [95% CI, 0.70–0.72]) and was substantially outperformed by NIHSS alone (*c*-statistic 0.82 [95% CI, 0.81–.83]). Adding the demographic and clinical variables from the clinical risk model to NIHSS produced only small improvement in discrimination beyond that obtained with NIHSS alone (*c*-statistic 0.84 [95% CI, 0.84–0.85], difference 0.02, 95% CI, 0.02–0.03, *P*<0.0001).

**Table 4.** NIHSS Discrimination of 30-Day Mortality Risk in Clinically Relevant Subgroups

Patient Subgroups	Chi-Square for 30-Day Mortality	*c*-Statistic (95% Confidence Intervals)
Age <80 y (N=17020)	2539.4	0.82 (0.81–0.83)

Age ≥ 80 y (N=16080)	3558.7	0.81 (0.80–0.82)

Men (N=14417)	2588.1	0.81 (0.79–0.82)

Women (N=18683)	3913.6	0.83 (0.82–0.83)

Prior stroke/TIA (N=9886)	1820.2	0.81 (0.79–0.82)

No prior stroke/TIA (N=23047)	4720.0	0.82 (0.82–0.83)

NIHSS indicates National Institutes of Health Stroke Scale; TIA, transient ischemic attack.

NIHSS analyzed as a continuous variable.

## Discussion

In assessing the relation of the NIHSS to 30-day mortality after acute ischemic stroke among GWTG-Stroke Medicare beneficiaries, this study finds that NIHSS is a very strong discriminator of mortality risk. There is a graded near-linear relationship between first recorded NIHSS and higher 30-day mortality risk. This study also demonstrates that with categorization of NIHSS into 3 or 4 groups, acute ischemic stroke patients can be readily identified as being at low, medium, or high risk for 30-day mortality, even in the absence of any other demographic or clinical variables. NIHSS provided excellent discrimination of 30-day mortality among multiple clinically relevant subgroups. NIHSS as a continuous variable or in categories resulted in far better discrimination of 30-day mortality risk then a clinical model not including stroke severity. These findings highlight the importance of a valid specific measure of stroke severity as a determinate of mortality after acute ischemic stroke for Medicare beneficiaries. Further, this study suggests that it may be vital for optimal discrimination to include stroke severity for risk stratification and risk adjustment in acute ischemic stroke.

The NIHSS was developed and subsequently validated as a tool for assessing the initial stroke severity.^20,21^ It has subsequently been shown to be predictive of a variety of stroke functional outcomes.^7–10^ Stroke severity as indexed by NIHSS has also been shown to be predictive of mortality after acute ischemic stroke.^9–14^ One study of 360 ischemic stroke patients admitted to a single hospital in Taiwan identified admission stroke severity as measured by NIHSS score as the strongest predictor of 3-month mortality, with an odds ratio of 1.17 (95% CI, 1.12–1.22) per point.^12^ Another study analyzed 479 patients admitted to a single center in Switzerland, advanced age, and high NIHSS were the only independent predictor of 30-day mortality.^11^ A study from 7 centers in Germany found NIHSS obtained within the first 6 hours of admission to be highly predictive of 100-day survival (*c*-statistic 0.86).^13^ A prior study for GWTG-Stroke showed that NIHSS was the strongest predictive variable for inhospital mortality and substantially improved the performance of a model based on clinical variables without stroke severity (*c*-statistic improved from 0.72 to 0.85).^14^ However, these studies were either small, drawn from select patients enrolled in clinical trials, conducted in a small number of centers outside the United States, or were confined to inhospital mortality. The findings from the present study, drawn from hundreds of hospitals from all regions of the United States and tens of thousands of patients, substantially extend these prior findings with follow-up to a standard 30-day outcome, and may be viewed as having greater external validity.

In the present study, a model with NIHSS alone without other variables had a *c*-statistic of 0.82, a range where clinical risk models are regarded to have genuine clinical utility for individual decision making.^22^ This illustrates the very strong relation between stroke severity and 30-day mortality. The mortality risk discrimination capability of NIHSS, either in a continuous or categorical variable, among Medicare beneficiaries with acute ischemic stroke compares very favorably to other mortality prediction models incorporating the NIHSS or other clinical measures of stroke severity (previously reported *c*-statistics 0.79 to 0.86) and appear superior to models with demographic/clinical variables without severity (previously reported *c*-statistics 0.69 to 0.75).^13,14,16,23^ Measures of stroke severity appear to be vital for optimal discrimination of mortality risk.

NIHSS was recorded in 37% of hospitalized acute ischemic stroke patients during the time frame of this study. The time and expertise needed to perform even a short standardized stroke severity assessment is an important barrier that will need to be further overcome. Recent data suggest that the NIHSS is being documented more frequently. In the first 2 quarters of 2011 in GWTG-Stroke, NIHSS was recorded in 61.1% of acute ischemic stroke patients (data on file, Duke Clinical Research Institute (DCRI)). Further study will be needed to determine the best methods for ensuring high rates of use of the NIHSS in routine clinical practice or whether other simpler measures of stroke severity could be developed and substituted without substantial loss of prognostic information.

### Limitations

There are several limitations in this study to consider. This study includes only patients in fee-for-service Medicare and thus does not include patients who were enrolled in managed care, the uninsured, and patients younger than 65 years of age. The relationship between NIHSS and 30-day mortality may differ among younger patients and those with different type of insurance. However, it is Medicare beneficiaries with acute ischemic stroke for whom 30-day mortality rates that are being considered for public reporting by CMS. The hospitals studied are those participating in GWTG-Stroke and may not be representative of all hospitals caring for patients with acute ischemic stroke. Although hospital characteristics for GWTG-Stroke differ from the nation, the demographics and clinical characteristics of Medicare beneficiaries in GWTG-Stroke have been demonstrated to be very similar to the overall Medicare acute ischemic stroke patient population.^24^ The analysis includes only patients with NIHSS documented, which may introduce selection bias, although differences in patient characteristic and 30-day mortality of patients with or without NIHSS documented are small. Further the demographics, characteristics, and 30-day mortality rates of patients with NIHSS in this study were similar to the overall Medicare acute ischemic stroke population, suggesting these findings may have external validity.^24^ Study data were collected on the basis of the medical record and depend on the accuracy and completeness of clinical documentation and chart abstraction. The NIHSS was analyzed as assessed and documented in clinical practice, the precise timing of NIHSS assessment was not collected, and the intra- and interobserver reliability at participating hospitals was not evaluated. Participating hospitals were encouraged to assess NIHSS prospectively. However, it is possible that, for some patients, NIHSS was determined retrospectively on the basis of the documented neurological examination; though this approach has been shown to produce reliable and valid results.^25^ Any imprecision and lack of uniformity in scoring would be expected to diminish, not enhance, the prognostic value of NIHSS. We did not evaluate other metrics of stroke severity. We also did not assess other important outcomes that may be predicted by NIHSS including health-related quality of life, disability, patient satisfaction, rehospitalization, or other clinical outcomes that may be of interest at 30 days or at longer time intervals after stroke.

In summary, the NIHSS provides substantial prognostic information regarding mortality within the first 30 days among Medicare beneficiaries with acute ischemic stroke. This index of stroke severity is a very strong discriminator of mortality risk whether evaluated as a continuous or categorical risk determinate. With categorization of NIHSS into 3 or 4 groups, Medicare beneficiaries with acute ischemic stroke can be readily identified as being at low, medium, and high risk for 30-day mortality. These findings suggest that it may be critical to collect and include stroke severity for optimal risk stratification and risk adjustment of 30-day mortality for Medicare beneficiaries with acute ischemic stroke.
